# The organizational challenges in the management of the revised national tuberculosis control program of India: an overview

**DOI:** 10.11604/pamj.2020.36.213.16501

**Published:** 2020-07-24

**Authors:** Sankalp Yadav, Gautam Rawal

**Affiliations:** 1 Department of Medicine and Tuberculosis, Chest Clinic Moti Nagar, New Delhi, India,; 2 Department of Respiratory Intensive Care, Max Super Specialty Hospital, New Delhi, India

**Keywords:** Directly observed treatment, short-course, tuberculosis, Revised National Tuberculosis Control Program, awareness

## Abstract

The developing world is facing a serious problem of tuberculosis (TB) since ages. The condition is really profound in resource-constrained countries. The situation in some of the high TB burden countries is so grave that there are huge numbers of TB patients and deaths due to TB. TB control in most of the countries is done by the national TB control programs. In India, this is popularly known as the Revised National Tuberculosis Control Program (RNTCP). The RNTCP comes under the umbrella of the national health mission. The main components of RNTCP are directly observed treatment, short-course (DOTS) and DOTS-Plus. The effective and adequate implementation of the RNTCP is the most effective solution to control the ever growing cases of TB. The present situation, as detailed in the WHO global annual TB report, with ever-rising cases of various categories of TB is really scary and demands prompt attention. In this paper, the authors highlight the important issues related to the RNTCP in India. The main motto of writing this paper was to address the challenges associated with the organizational structure of the national TB control program of India and to suggest solutions for the same. The authors believe that these challenges could pose a serious threat to the efforts aimed at TB elimination from India. Besides, this paper will serve as a tool to modify and/or to formulate new guidelines for the betterment of the program. Also, the challenges detailed here are usually common in the other high TB burden countries of the world and this will help the program managers worldwide.

## Essay

TB is known for ages and has become a global public health problem [1]. The recent reports from the WHO wherein it shows that the “STOP TB” goal has been achieved is a ray of hope for the long-term goal of eliminating the TB from the face of the world by 2050 and from India by 2025 [1,2]. The Revised National Tuberculosis Control Program (RNTCP) was launched in the year 1997 [1,2]. India's RNTCP has made significant progress in TB control over the last decade through countrywide DOTS implementation [3,4]. India accounts for about one-fifth of the global burden of TB. The DOTS-based RNTCP has been remarkably successful in achieving the global targets of detecting 70% of the estimated TB cases and curing 85% of them [3,4]. The RNTCP since its inclusion as a national TB control program has given good results, as evident by the detailed WHO global annual reports, yet the TB elimination looks a far-sighted dream [4,5]. There are a number of issues that are hampering the ongoing RNTCP progress in India. The major contributors to this could well be related to the ever-growing population; lack of staff; lack of funds; lack of dedication in the RNTCP staff; corruption; issues related to patients; improper monitoring and supervision, e.t.c. to name a few [1,6].

Every year the world TB day is celebrated on the 24^th^ of March and the reports of multiple Advocacy, Communication and Social Mobilization (ACSM) activities from all over the country are made available [7]. However, such activities are short lasting and they disappear soon after the 24^th^ of March [7]. The importance of awareness in TB control is well emphasized elsewhere [7]. The awareness is absolutely important for the control of TB in a fast multiplying population. The role of such activities also gains an important role, especially in countries where the health expenditure both by the government and the general public at large is meager [7]. In the resource-constrained countries where the per capita income is low, the lay public is less concerned for any disease control or elimination, be it TB [7]. In a society where 40% of the population could not arrange for food two times a day, problems like TB remains unheeded TB [7]. Besides, the TB control was an important part of Millennium Development Goals (MDG) (now Sustainable Development Goals) and health for all [1]. In this write-up, the authors discuss the problem associated with the RNTCP at the grassroots level and also provide possible solutions for these issues for the effective and efficient implementation of the RNTCP targeted at TB elimination by 2025 [2].

**The problems of RNTCP:** there are a number of issues impeding the desired outcomes of the RNTCP. The whole program is well designed and aimed at TB control, but the elimination of TB at this stage is difficult unless major changes are made for smooth implementation of the RNTCP. Some of these issues are:

**Issues due to the geographic location of the district tuberculosis center (DTC):** the RNTCP guidelines clearly state that chest symptomatic patients with at least two or more than two weeks cough should undergo sputum examination [8]. The problem arises when the patient has to go to the Designated Microscopy Center (DMC) for the sputum examination slip and sputum containers. Many a time the sputum containers are provided to the patients, but no details about the timing and the way to produce the best quality sputum are explained. This leads to poor quality sputum samples and thus a number of times the diagnosis of TB is missed/delayed [8]. Also, it has been noticed that the chest symptomatic patients are first referred to the DTC from the DMC´s and the sputum examination is done once the medical officer-TB (MO-TB) at the DTC advice the same. The whole process is really tiring for a patient who has to travel long distances from his residence to the DTC and then again from the DTC to the local DMC. In fact in the majority of cases, the same patient has to travel again the next day with an early morning sample to the same DMC for submission. Although the RNTCP clearly mentions that any TB suspect or chest symptomatic can get his/her sputum examination done from any DMC, but often the lab technicians (LT) discourages such things [9]. Sometimes the poor patient lacks adequate funds to commute and thus they skip these tiring efforts, thereby becoming a potential source of TB spread to the society [1,8]. Also, the daily wagers who are paid hourly find it near impossible to skip wages for these free yet tiring investigations and thus become “diagnostic defaulters” when they fail to submit the necessary number of sputum samples at the DMC and those who do not come back for results, are lost to treatment and follow up [8].

**Issues related to lab technicians (LT):** the LT is posted at each DMC for the sputum microscopy [9]. It has been noticed that some of these LT´s do not accept the sputum especially when a government holiday is scheduled the next day. Also, the refusal to take sputum samples has also been reported, if the patient reaches late on the day of submission. The malpractice of asking the patients to bring an address proof before submission of the sputum sample has also been noted from a number of DMC´s. Besides, rude behavior is a common problem. The correct way of producing a good sputum sample is not explained to the patients, who provide saliva for the investigation thus affecting the overall diagnosis and management [10]. Also, most of these DMC´s are far from the DTC and thus in the absence of daily monitoring many a time these LT´s are missing from their duties ultimately affecting the RNTCP as a whole. Large numbers of samples, all combined together often reduce the observation time per slide to less than 60 seconds and this also contributes to a reduction in the sensitivity of the test [8]. Also, during the Random Blinded Re-Checking (RBRC) a number of slides marked negative by the LT´s comes out to be positive and the vice versa thus questioning the overall commitment of the LT´s [11].

**Issues related to the lack of staff:** the performance of healthcare systems is directly proportional to the numbers, distribution, knowledge, skills and motivation of its workforce, particularly of those individuals who are responsible for delivering the services [12]. In 2003, national TB program (NTP) managers from 18 of the 22 TB high-burden countries (countries that together account for approximately 80% of the global TB burden), ranked inadequate human resources for health (HRH) first within the top five constraints to reaching the WHO global TB control targets [12]. The same situation exists in RNTCP in India [12] and the staff that is currently employed is also mostly contractual and thus there are issues related to inadequate pay; long working hours; requirement of a greater degree of commitment; job dissatisfaction; depression e.t.c. [13,14]. The contractual staff is made to work six to eight hours and are paid a meager amount without any remarkable perks and allowances [14]. This leads to a careless attitude and lack of commitment and dedication [14,15]. These staffs are also devoid of social security cover under different legislative provisions, such as the Employees' Provident Fund Act 1952, the Employees' State Insurance Act 1948, the Workmen's Compensation Act 1923 and the Maternity Benefit Act 1961 [14]. There are issues related to delayed payment of salaries and non-payment of arrears which are very common [16]. Every year lots of funds are given to the RNTCP, but the staff is never made permanent. This results in professional jealousy among the staff, as the maximum staff is contractual, but at the same time, they are working in the premises where the permanent staff employed by the state or national governments are also working. There are no perks in the salaries and thus basically there is no incentive to work efficiently [14]. Many a time these RNTCP staffs are also involved in other jobs or employed elsewhere as a daily wager and thus they are often missing from their workplaces since they have to attend their add-on jobs. The amount of money that they get from RNTCP as a salary is not sufficient and thus they are compelled to work in other places in order to meet their ends. Lack of HR strategies, inadequate HR planning and management, poor deployment practices, inflexible contracting arrangements and inability to create new posts or increase salaries resulting from national/international regulations capping social sector spending have contributed to the HRH crisis in RNTCP [12]. Besides, this has increased pressure on health systems and led to the death and disability of the existing workforce itself [12]. Disease-specific programs (including TB) are still struggling to meet their targets and governments and their financial/technical partners have finally recognized this is largely due to shortcomings in the health care workforce [12].

**Issues related to senior TB lab supervisors (STLS):** the STLS´s are an important part of the RNTCP and the proper monitoring and supervision of the DMC´s is their main role [9]. There are certain issues related to STLS as well. The STLS´s are many a time casual in their approach towards their roles and do not perform their duties as per the guidelines. They leave the DTC with permission to supervise the DMC´s for on-site evaluations (OSE), but they do not go to these DMC´s. Also, the favoritism and lack of attention to the work by LT´s or blind faith in the LT´s is a common problem. The inadequate and improper supervision has affected the desired results of the RNTCP. The STLS and around 20-50% of LT´s under RNTCP are contractual positions and one of the major criteria for their annual contract renewal is based on RBRC performance [17]. Hence, there may be a possibility of nexus among these contractual workers to bury the true findings [17]. Blinding of RBRC slides which are the responsibility of the DTO may not be strictly followed at all DTC´s [17].

**Issue related to senior treatment supervisors (STS):** STS´s are also equally important for supervising the DOT centers and coordinating with the DOT providers [9]. The STS has also been found to be missing from their duties. The lack of attention towards the work of DOT providers and blind faith in them are common problems. A number of times the STS are not even aware of the absence of the DOT provider from the DOT centers. This results in panic and confusion among the patients. The TB patients are already in poor shape due to their disease and thus if the medicines are not provided to them daily then it may lead to instilling fear and panic in them. The reasons could be many, but the one that is worth mentioning, in this case, is the shortage of STS in the program [12]. There are centers where one STS is supervising more than 13 DOT centers with the population well above the set criteria.

**Issues related to DOT providers:** the issue with the DOT providers is more or less similar to other categories and are related to lack of staff, overwork, underpaid work and lack of commitment towards their jobs [12]. The rude behavior and casual approach towards work are common. Delay in starting the treatment within the time frame as per the guidelines is common [8,12]. The issue of giving multiple strips of the anti-TB medicines to the patients is also not uncommon. It has been noted that in some remote areas even selling of the whole patient wise boxes is practiced. The lack of availability of properly maintained inventory, movement registers e.t.c. are also common. Inadequate and improper attention to the weight of the patients, fasting blood sugar levels, HIV status e.t.c. have also been noticed at some DOT centers and the ugliest part is when a patient has been lost to follow-up and either to keep good repute with the seniors or to avoid unnecessary cross-questioning, the DOT provider continues their treatment on paper as a normal ongoing case thus deliberately misleading the whole program.

**Issues related to lack of supervision from the district tuberculosis officer (DTO):** the DTC is headed by the DTO [7,9]. DTO is responsible for the proper implementation and running of the RNTCP in his district [7,9]. To ensure this the DTO is supposed to visit his DOT centers and DMC´s regularly [9]. But it has been observed that the DTO´s are irregular in their monitoring and supervision. Besides, the DTO is also responsible for the administrative works related to the district and thus is so absorbed with these works that the monitoring and supervision take a back step. Moreover, the high burden centers are affected really badly due to this lack of adequate monitoring and supervision. The lack of vehicle and funds to procure the same has also been noted both in rural and urban areas. Thus, affecting the overall surveillance and monitoring.

**Issues related to MO-TB:** the MO-TB at the DTC is also required to visit the DOT centers and DMC´s regularly as per the RNTCP guidelines [9]. But many a time this is missing. A number of factors may be responsible for this. The most important of these is the lack of staff [12]. Most of the time there is only one MO-TB per DTC. The single MO-TB has to do the daily OPD of the new and old TB patients. There is a very high number of old and new cases in the OPD. Besides, the other patients with chest diseases also contribute significantly to daily OPD. Then the same MO-TB has to supervise his centers. Mostly these MO-TB´s are contractual and are not paid adequate salaries [16]. The delay in payment of salaries is very common [16]. In such a situation the role of regular monitoring sometimes takes a back step. Besides, the lack of attention towards the problems by the higher authorities often leads to distrust and dissatisfaction with the job. There is no encouragement in terms of incentives or perks in the salaries of the contractual staff [14] and the unequal salaries among the doctors who are permanent and those who are contractual may result in frustration and thus may affect the RNTCP adversely [12,14]. Also, these contractual doctors are given contracts for the stipulated time and thus there is no job security and thus having a huge impact on the general attitudes of the MO-TB [12].

**Issues related to drug supply and storage:** a number of times it has been noted in the past that some drugs are not available and thus directly affecting the poor patients [18]. The delay in the supply of drugs is common in the cases of TB. One of the major strategies in RNTCP´s DOTS is the “uninterrupted drug supply” [9]. The improper maintenance of the adequate supply of drugs is a major contributor to instilling the fear among the TB cases [1,18]. This issue arises mostly at the level of the state and above who are responsible for the uninterrupted supply of drugs. Besides, the storage of drugs is also not as per the guidelines at many DTC and DOT centers [19]. There are issues with electricity, humidity/moisture control, pests e.t.c. Majority of metro cities in India lack the proper drug storage facility, the condition in remote areas is even graver [19].

**Issue related to poor quality of TB drugs:** a number of studies reported that many drugs including the antimalarial drugs were having sub-therapeutic or no levels of drugs at all [1]. Thus there is a possibility of the role of spurious drugs in the RNTCP which can lead to inadequate treatment or treatment failure [1,19]. Besides, this will also result in the development of drug-resistant strains of the bacilli [1]. The problem of spurious drugs is looming large over the Indian markets and even TB patients are not spared [1,20]. The lack of strict laws and the improper or no enforcement of the existing laws and the lackluster attitude of the regulating bodies with rampant corruption may adversely affect the RNTCP [20]. Quality assurance remains dubious especially in developing countries [20].

**Issues related to the improper TB notification:** TB has been a notifiable disease since 7^th^ May 2012 [6]. The private practitioners are required to notify all the TB cases [6]. However, the same is not happening as desired. There is a lack of commitment of the private practitioners in notifying their cases. Thus, the actual figure of TB cases in an area is not always the total cases present in the area. The STS are required to visit their TB units regularly and are supposed to provide the line list of all the private practitioners [9], but the same is not always possible due to poor attitude and commitment from the side of the STS. Unless all the TB cases are notified we cannot come to the conclusions about the TB control in India. Besides, the unregulated private anti-TB treatment leads to wrong diagnosis and treatment and has been explained in detail elsewhere [1].

**Issues related to unhygienic working conditions:** there is one more issue which is a very big hurdle in the RNTCP smooth functioning. The issue relates to the unhygienic workplaces. Most of the DOT centers and DMC´s in New Delhi are not having any provision of proper ventilation. Some chest clinics are not even having basic infection control facilities and thus the life of doctors and other staff is always at risk of catching the infection from the TB bacilli. Also, the condition is so pathetic at certain places that the water supply, electricity, and furniture´s like chairs and tables are also missing. An unsafe health facility environment such as unsuitable furniture´s, inappropriate lighting, poorly designed workstations, excessive noise, lack of ventilation, poor supervisor support, poor workspace, poor communication, poor fire safety measures for emergencies and lack of personal protective equipment, can adversely affect the productivity of the employee [21]. The role of hard-working staff who is working under such highly infective settings is commendable, but at the same time, no efforts are made to solve this issue. The policymakers should seriously look into this issue as to expect the best results from RNTCP implementation in the absence of adequate facilities to the staff is very difficult.

**Possible solutions:** the problems highlighted in this paper are only a tip of the iceberg and there are many other issues impeding the success desired for the RNTCP. The above-mentioned issues can be addressed by the involvement of all the stakeholders like the governments, RNTCP staff and patients. The government should ensure that the problems related to overwork and underpaid work resulting in job dissatisfaction in the RNTCP staff should be addressed. The contractual staff should be made permanent as this will help in increasing the commitment towards their duties. Also, if the same cannot be done, then at least the pay scale and perks of contractual staff should be on par with the permanent staff. The issues arising due to lack of commitment need to be solved. The DTO should discuss the issues with the staff and thus regular staff meetings are important in solving the issues of the staff. The regular counseling of the staff, stress management and efforts aimed at solving their issues should be given prime importance. The improvement in the workplace environment with basic facilities like water and electricity should be mandatory. TB notification should be given prime importance and strict rules and regulations should be drafted so that each private practitioner should notify his TB case [6]. The RNTCP can help us achieve the TB free India, but the dissatisfied staff implementing it and other ground realities are the barriers to eliminating the TB.

**Conclusion:** the major challenges hampering the success of the RNTCP need to be addressed. The issues are at every front, but these problems can be efficiently dealt with. The government should revamp the whole system of supervision and monitoring. Efforts should be put into active and thorough surveillance. The total number of evaluations should be increased and accountability given to each and every member involved in the program should be supervised timely. The country gets enough funding from various agencies and this needs to be properly used. Mere emphasis on getting desirable reports on paper and lack of attention towards the problems related to the service providers and service recipients will not help in the long run. The problem of TB is a public health issue and a major cause of morbidity and mortality and thus prompt efforts to make the whole program effective at the ground level is imperative.
